# Immunological Tolerance and Function: Associations Between Intestinal Bacteria, Probiotics, Prebiotics, and Phages

**DOI:** 10.3389/fimmu.2018.02240

**Published:** 2018-10-09

**Authors:** Luis Vitetta, Gemma Vitetta, Sean Hall

**Affiliations:** ^1^Discipline of Pharmacology, Faculty of Medicine and Health, School of Medicine, The University of Sydney, Camperdown, NSW, Australia; ^2^Medlab Clinical Ltd., Sydney, NSW, Australia

**Keywords:** immunological tolerance, probiotics, prebiotics, *Lactobacilli*, *Bifidobacteria*, inflammation, bacteriophages

## Abstract

Post-birth there is a bacterial assault on all mucosal surfaces. The intestinal microbiome is an important participant in health and disease. The pattern of composition and concentration of the intestinal microbiome varies greatly. Therefore, achieving immunological tolerance in the first 3–4 years of life is critical for maintaining health throughout a lifetime. Probiotic bacteria are organisms that afford beneficial health effects to the host and in certain instances may protect against the development of disease. The potential benefits of modifying the composition of the intestinal microbial cohort for therapeutic benefit is evident in the use in high risks groups such as premature infants, children receiving antibiotics, rotavirus infections in non-vaccinated children and traveler's diarrhea in adults. Probiotics and prebiotics are postulated to have immunomodulating capabilities by influencing the intestinal microbial cohort and dampening the activity of pathobiont intestinal microbes, such as *Klebsiella pneumonia* and *Clostridia perfringens*. *Lactobacilli* and *Bifidobacteria* are examples of probiotics found in the large intestine and so far, the benefits afforded to probiotics have varied in efficacy. Most likely the efficacy of probiotic bacteria has a multifactorial dependency, namely on a number of factors that include agents used, the dose, the pattern of dosing, and the characteristics of the host and the underlying luminal microbial environment and the activity of bacteriophages. Bacteriophages, are small viruses that infect and lyse intestinal bacteria. As such it can be posited that these viruses display an effective local protective control mechanism for the intestinal barrier against commensal pathobionts that indirectly may assist the host in controlling bacterial concentrations in the gut. A co-operative activity may be envisaged between the intestinal epithelia, mucosal immunity and the activity of bacteriophages to eliminate pathobiots, highlighting the potential role of bacteriophages in assisting with maintaining intestinal homeostasis. Hence bacteriophage local control of inflammation and immune responses may be an additional immunological defense mechanism that exploits bacteriophage–mucin glycoprotein interactions that controls bacterial diversity and abundance in the mucin layers of the gut. Moreover, and importantly the efficacy of probiotics may be dependent on the symbiotic incorporation of prebiotics, and the abundance and diversity of the intestinal microbiome encountered. The virome may be an important factor that determines the efficacy of some probiotic formulations.

## Historical perspective—microorganisms and immunity

The mid seventeenth century ushered the beginning of a new era in medicine with the discovery of a microscopic world of bacteria and fungi (1) that in the twenty-first century these entities have been demonstrated to play a crucial role in shaping human immunity and maintaining immunological and metabolic tolerance throughout a lifetime (2, 3).

The history of health assertions relative to live microorganisms present in food is extensive and predates antiquity. Particularly, that involving lactic acid producing bacteria as described in the Old Testament from Persian scriptures where it was alluded to, that Abraham's consumption of sour milk provided a health and longevity benefit (4). Furthermore, writings from the Roman historian *Plinius* from the first-century, suggested that the administration of fermented milk foods could be used to treat gastroenteritis (4). Microbiological observations (5–7) and Metchnikoff (7) are credited with promoting the extensive health effects and restorative shifts in the balance of the gastrointestinal microbial cohort with the consumption of lactic acid bacteria. Metchnikoff who was at the Pasteur Institute, suggested that an extension of the life span of Bulgarian workers resulted from the ingestion of fermented milk foods (7). The consequence of consuming *Lactobacilli* inoculated yogurts could lead to a significant decrease of intestinal toxin-producing bacteria and that this effect then led to an increase in longevity. Moreover, Tissier (5, 8) reported that *Bifidobacteria* predominated in the intestines of breast-fed infants (5) and subsequently endorsed the administration of *Bifidobacteria* to infants distressed with diarrhea, and then further suggested that *Bifidobacteria* superseded the putrefactive gut pathobionts that would cause disease (8).

The early studies on lactic acid bacteria became recognized as the link between microbiology and the foundation of immunology (9). Metchnikoff's immunological studies credit his discovery of phagocytosis by macrophages and microphages as the elucidative step in host-defense mechanisms that established innate immunity; simultaneously Ehrlich defined the side-chain theory of antibody formation and the immunological pathways of how antibodies counteract toxins that encourage bacterial lysis. Furthermore, Erhlich also documented on how with the participation of complement, it led to an enhanced understanding of humoral adaptive immunity (9).

The early studies on lactic acid bacteria became recognized as the link between microbiology and the foundation of immunology (10). Metchnikoff's immunological studies associated with phagocytosis with macrophages and microphages resulted as an important discovery step in host-defense mechanisms. These studies established the foundation of cell innate immunity. Simultaneously Ehrlich explained the side-chain model of antibody formation and the immunological pathways of how antibodies counteract systemic toxins and encourage bacterial lysis. Furthermore, Ehrlich proposed that the participation of complement enhances the understanding of humoral adaptive immunity (10). Recent advances describe an overview of immunity as divided into two predominant systems that are determined by the speed and specificity of the reactions that occur (11). That is innate immunity describes chemical, physical, and microbiological barriers that usually encompass immune system elements of neutrophils, monocytes, macrophages, and the complement network of cytokine proteins and other acute phase proteins that provide an immediate response to an infective insult, a response that is imperative for survival. Cell mediated immunity is the immune characteristic exhibited by higher animals that comprises antigen specific responses through T and B lymphocytes, a slow but precise response to an infective agent (12). Moreover, a link has been recognized to exist between the innate and cell mediated immunity systems that is necessitated to complete the immune response and that is the role of complement that effectively bridges both systems in order to neutralize an infective bacterial, viral or other insult (13). The human microbiome project (14) has redefined the role of bacteria that live on and within humans especially in the intestinal tract, from one that was considered to be a site of toxic waste and pathogenic bacteria to a site with important immunological activity (15). Recently, reviews have described how the role of probiotics enhancing immunological functions in the intestines to restore local and extra-intestinal mucosal and innate immunity equilibrium have gained significant support (16, 17).

## Dysbiosis

A historical perspective on the origin of the term “dysbiosis” has been recently documented (18), with Haene (19) a German microecologist, credited with popularizing the term (18). Moreover, Haenel was also instrumental in contrasting dysbiosis with eubiosis which he referred to this latter term as the “normal state” (19).

Dysbiosis usually refers to an imbalance of intestinal bacteria that occupy the lumen of the gut that refers to adverse microbiome pattern shifts that have been associated with disease development and progression (18). Further, dysbiosis has been reported to be the subject of multiple explicit and semi-explicit delineations (18).

The chronic diseases that have been associated with intestinal microbiome dysbiosis include intestinal inflammatory diseases (e.g., Ulcerative colitis, Irritable Bowel Syndrome), auto-immune diseases (e.g., multiple sclerosis, asthma), metabolic diseases (e.g., diabetes), neuro-degenerative diseases (e.g., Parkinson's Disease, Dementias), and cancer (20). The community of bacteria that inhabit the intestines has been investigated from multi-dimensional studies with samples from different geographical areas. The samples were constructed from variations observed in the concentration of three dominant/conserved bacterial genera namely, *Bacteroides* (enterotype 1); *Prevotella* (enterotype 2); and *Ruminococcus* (enterotype 3) (21, 22). The topographical view of the intestinal microbiome has provided the basis to further understand the bacterial phyla configurations associated with dysbiotic shifts that have been correlated with disease states. As for example, when comparing the intestinal microbiome of obese to lean subjects studies have shown increased phyla abundances in *Firmicutes* and decreased *Bacteriodetes* (23).

## Intestinal bacteria and immunity

The human microbiome project has largely changed the way bacteria have been viewed in the large bowel; from a collection of waste products and pathogens to a more pragmatic view concerned with early immunological and metabolic development and to sustain a stable equilibrium. The mucosal surfaces of the intestines provides a large complex and interactive surface area between the commensal bacterial cohort and the intestinal epithelia (24). Specifically, the complex nature of the intestinal architecture is evident from the components of the differentiated epithelial cell types that are encountered such as enterocytes (approximate turnover of 3–5 days which migrate out of the aberrant crypt), enteroendocrine cells, goblet cells, tuft cells, and Paneth cells (approximate turnover of 30 days which do not migrate out of the aberrant crypt) (25, 26). The cross-talk that has evolved between bacteria and host gut tissue has spanned millennia (16). In particular, Paneth cells synthesize and secrete proteins and antimicrobial peptides (i.e., α/β defensins; cathelicidin; 14β-glycosidases; C-type lectins; ribonuclease), activities that emanate from various external and internal stimuli (e.g., intestinal bacterial milieu such as bacterial surface components) and toll-like receptor activity (27).

**Figure 1:**
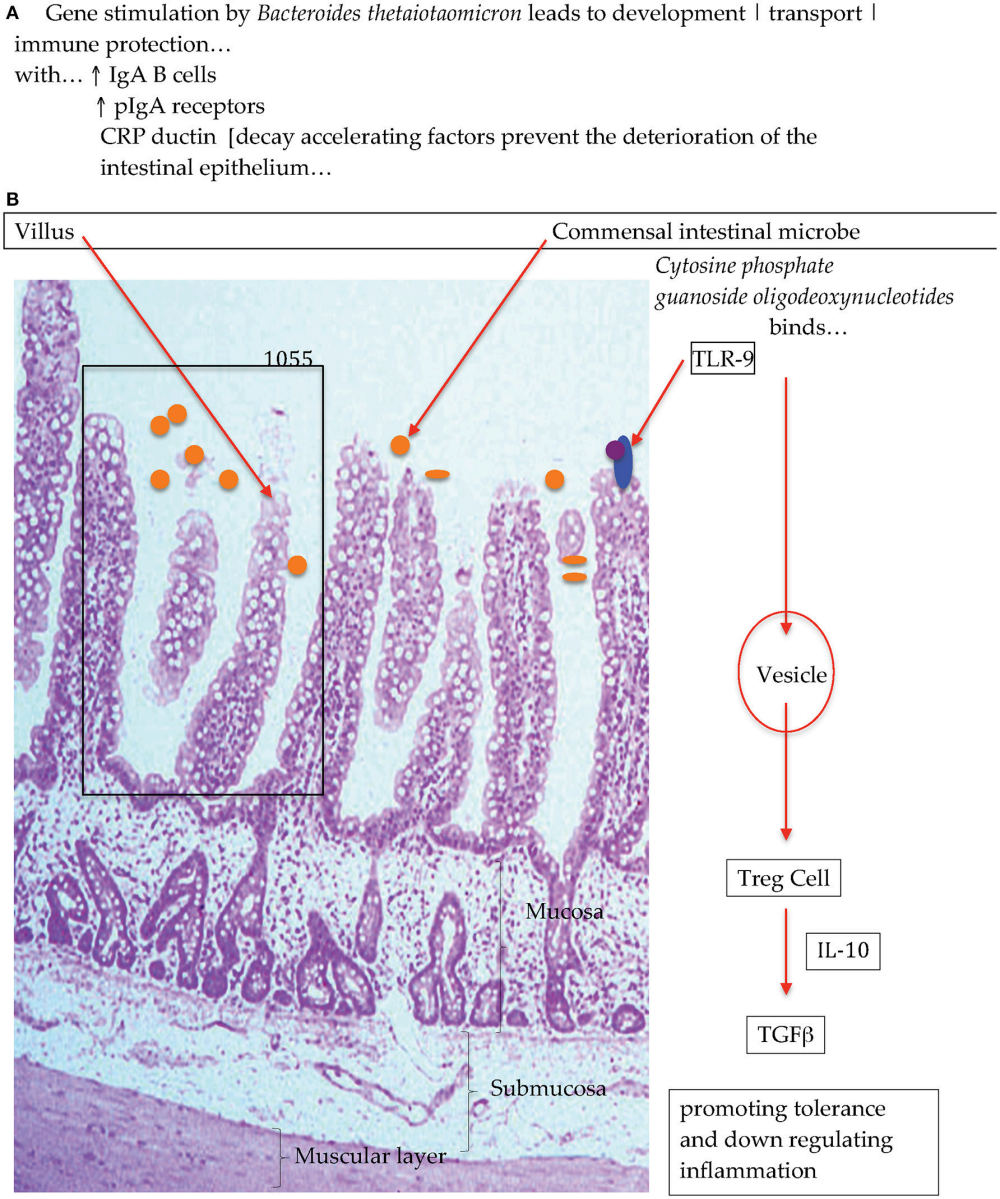
Diagrammatic representation of **(A)** bacterial colonization stimulates intestinal gene activation and **(B)** commensal bacteria interactions with Toll-Like Receptors to promote immunological equilibrirum (28).

Experiments with gnotobiotic murine models have shown that when colonizing the animals with a single bacterium that adhered to the surface of the intestinal epithelia encouraged the growth and activity of a set of genes in the host animal, that were involved in immune function, and the elaboration of proteins that protected against the deterioration of the gut epithelia [Figure 1; (28, 29)] Additional experiments have also reported on the interactions between commensal bacteria and host immune cells and have shown that macrophages that are in close proximity to the base of the intestinal epithelia participate in antigen recognition and responses (30). That is, macrophages/dendritic cells take up antigen and produce cytokines and depending on which cytokine is produced, a response is elicited such as anti-inflammatory. Research has advanced the idea that antigens from the intestines or from the environment are transcytosed (31, 32) by specialized enterocytes namely, Microfold-cells into the sub-epithelial dome region of the Peyer's patch mucosa, an area rich in macrophages*/*dendritic cells (33–35) where these antigen-presenting cells reside and progress to collect the trancytosed bacteria and macromolecular antigens (36). In the *lamina propria* macrophages have been demonstrated to sample antigens, entero-pathobionts and commensal bacteria through trans-epithelial dendrites. Intestine-resident macrophages (CX3 CR1hi^+^) that are resultant from blood monocytes (Ly6C^+^) do persist in close physical proximity whilst maintaining the integrity of the intestinal epithelial cells and barrier function. Experiments investigating specific effects with the human and animal commensal bacillus, *Clostridium butyricum*, have demonstrated that in a murine model the spore forming anaerobe induced IL-10 producing macrophages that suppressed an acute experimental inflammatory outcome (37). The explicit effect triggered by *Clostrodium butyricum* treatment showed that the outcome was not associated with cytokine IL-10 production by Treg cells but that instead the anti-inflammatory effect was due to IL-10-producing F4/80+CD11b+CD11c^int^ macrophages through the TLR2/MyD88 pathway and subsequently observed to have accumulated in the inflamed mucosa. The posit that macrophage sampling eliciting and then escalating an antigen specific immune response has recently been insightfully progressed (38, 39). Man et al. (38) in a murine study with *Salmonella typhimurium* demonstrated that there also exist protective mechanisms in the intestines that hinder the access of bacteria to the intestinal epithelia that thereby block pathogen penetration across the intestinal barrier. Specifically, intestinal epithelium cell emerging signals, trigger the intraluminal migration of CX3CR1^+^ cells forming an intricate network of cellular defense. This protective effect presents an additional barrier to the copious amounts of mucus and Immunoglobulin A that are constantly produced to maintain local homeostasis, when these first line of defense are breached the complex network of intraluminal migration prevents early local infectivity from progressing.

It is widely acknowledged and published in the scientific literature that food antigens do indeed affect immunological responses (40). In the intestinal induction of Tregs and Th17 is characteristic of the gut immune network (41). Consequently, the Th17 cells strengthen the mucosal barrier and concomitantly encourage intestinal epithelial cells to produce antimicrobial peptides, with the overall effect centered on maintaining local homeostasis (42). Regulatory T cells that promote tolerance do so to reduce reactivity to dietary and environmental antigens by decreasing intestinal inflammation. Strikingly this regulated pro-inflammatory to anti-inflammatory activity has been demonstrated to be a cooperative crosstalk between commensal bacteria, the host intestinal epithelia and the mucosal immune network to reduce the risk of inflammatory responses by down regulating the effect of dietary and or bacterial antigens that in turn maintains local immunity in equilibrium (43). For example the mucosal accumulation of Th17 cells has been shown to be subject to stimulation by commensal bacteria (i.e., Segmented Filamentous Bacterium) (44) indicating that commensal bacteria very much promote the accumulation of Th17 cells in the intestinal *lamina propria* (45, 46).

As a consequence, specific bacterial species have been shown to significantly influence immune tissue subsets of cells and maturation of innate immunity in health and disease [Table 1; (70, 71)]. Probiotic bacteria, in one instance, were posited to induce a re-regulation of the nuclear translocation of NFκB in intestinal epithelial cells by shaping the release of TNF-α that links this molecular action to a significant decrease in epithelial permeability and susceptibility to an inflammatory Crohn-like ileitis in the SAMP1/YitFc murine model that spontaneously progress to an inflammatory disease (72).

**Table 1:** Commensal intestinal bacteria and effects on the development of tolerogenic macrophages|dendritic cells; induction of Treg cells; and stimulation of TLRs.

**Intestinal bacteria**	**Immune tissue induction effects**	**References**
*Segmented Filamentous Bacteria* Gram positive, spore-forming obligate anaerobe	In the terminal ileum - Alter T cell subsets - Induce accumulation of Th17; induce serum amyloid A—a protein that acts on the *lamina propria* and stimulates Th17 inducing environment - Can drive an autoimmune disease	(47)
*Bacteroides fragilis* Gram negative, obligate anaerobe	In the large bowel [colon] (48) - Direct development of FoxP3+ Tregs - Bacterial *polysaccharide A* mediates conversion of CD4+T cells → FoxP3+Tregs - TLR2 signaling in T cells mediates this effect and not in macrophages|dendritic cells - *Polysaccharide* A–TLR2 ^−/−^ co-operations results in ↑ formation and release of IL−10 by Tregs and notably ↓the multiplication of Th17 cells in the intestines - PSA derived from this commensal is a symbiosis factor that promotes immunologic maturation within mammalian hosts	(49)
*Clostrodium cluster IV and Cluster XIVa*	Promote expansion of colonic and systemic Treg cells - Clostridia activate intestinal epithelial cells secretion of TGFβ and Treg inducing molecules MMP2/MMP9/MMP13/indoleamine 2,3-dioxygenase ↑ Tregs in the colon only- Clostridia in the intestines suggest an effect to multiple sites systemically - Tregs ↑ spleen/liver/lungs - Clostridia induction of Tregs PRRs - Mice deficient in MyD88/Rip2/Card9 have normal Tregs numbers in the colon - ↓ levels of systemic IgE/IL−4 and ↑ levels of ↑ levels of IL−10 producing splenocytes are found in experimental animal model of OVA–induced asthma in the presence of Clostridia	(50)
*Bacteroides thetaiotaomicron*	In the colon - Binding of immunoglobulin A, a requisite for *B. fragilis* (and other commensal intestinal bacteria) to occupy a defined mucosal niche in the intestines - Maintain host-microbe mutualism	(29) (51)
Lactic acid bacteria overall Lactic acid bacteria [most species] *Lactobacilli*|*Bifidobacteria* *L. rhamnosus GG* *L. casei* *L. reuteri* *L. paracasei* *L. plantarum* *L. acidophilus* *B. lactis* *B. bifidum* *B. infantis* *B. longum* *B. breve* *E. coli Nissle 1917*	Weak induction IL-12|TNF-α weak - Strong induction IL-12|TNF-α weak - ↑Treg cells producing ↑levels IL-10 (variable levels) - Increased FoxP3+ Tregs|suppressor activity - Modulate dendritic cell function - ↑FoxP3+ Tregs|TGF-β Tregs|TGF-β production - ↑FoxP3+ Tregs|IL-10|TGF-β production - Prime monocyte-derived dendritic cell|DC maturation dendritic cell|DC maturation |↑IL-10|↑FoxP3+ Tregs|activation of TLR-9 - ↑FoxP3+ Tregs|IL-10|TGF-β production - ↑FoxP3+ Tregs|IL-10|TGF-β production - ↑IL-10|↓IL-4|Treg-associated TGF-β production - ↑IL-10|↓IL-4|Treg-associated TGF-β production - Prime neonatal macrophages|dendritic cell - ↑FoxP3+ Tregs|↓TNF-α|IL-6 - ↑IL-10 production macrophages|DC - Activation TLR-2|dendritic cell maturation/activation|↑IL-10 production - ↑FoxP3+ Tregs	(52) (53) (54) (55) (56) (57) (58, 59) (60) (61) (62) (63) (64) (65) (66) (67) (68) (69) (10)

Among all the microbial metabolites expressed in the intestinal lumen the most abundant in the colon are SCFAs (73). Recently, probiotic species from the *Lactobacilli* and *Bifidobacteria* genera were demonstrated to increase the levels of fecal SCFAs (74). These chemical species exhibit multi-faceted regulatory roles in the local mucosal immune system in the gut. That is (i) SCFAs are a major energy source of intestinal epithelia significantly influencing gene expression (i.e., epigenetics) that is an important pre-requisite for maintaining the coherence of the epithelial lining and epithelium to epithelium tight junctions and mechanisms of defense. Hence it is envisaged that SCFAs can also (ii) regulate local mucosal derived innate immune cells such as macrophages or dendritic cells as well as neutrophils. Moreover, SCFAs also (iii) are involved in a bi-directional regulation of specific antigen-triggered adaptive immunity activities that are mediated by T and B lymphocytic cells.

## Probiotics

Probiotics were originally defined as live micro-organisms that could be added to fermented foods that in such a matrix could be advantageous to health by establishing an overall improved stability to the intestinal microbial cohort (75) and then modified by the FAO/WHO (76). Micro-organisms predominantly utilized as probiotics include various members of the *Lactobacilli* or *Bifidobacteria* species which are administered either individually or combinations of various formulations. The non-pathogenic yeast, *Saccharomyces boulardii*, has also been designated as a probiotic following its administration in both animal studies and human clinical trials. Further, the inability of probiotics to permanently colonize the intestines has let to posits that they be dispensed in sufficient quantities to maintain high amounts in the colon; and that probiotic species be of human origin.

### Probiotics: *in vitro* and laboratory animal data

The critical and important purported health-recommended effect of probiotics is the ability of these bacteria to enhance mucosal immune defenses (77). The gut associated lymphoid tissue can be distributed according to anatomical sites, with lymphocytes disseminated throughout (i) intestinal epithelia regions in contact with the *lamina propria* and (ii) structured lymphoid tissue sites, that includes Peyer's patches and mesenteric lymph nodes (35).

Studies with germ-free animals (gnotobiotic) clearly demonstrate that in the absence of significant intestinal microbial colonization, the effective component of the mucosal immune system remains immature, an outcome that enhances the host's susceptibility to bacterial infections by pathobionts (78). Moreover, general mechanisms for the function of probiotics have been associated with protective effects provided against pathobiont microbial colonization and translocation within the intestines (79). It has been postulated that mechanisms include production of antibiotic type substances (i.e., reuterin) (80) and competition for receptor sites on the mucosal intestinal surface (81). Other mechanisms such as heightening immune defenses of the host that produce adjuvant effects, amplified immunoglobulin A production and cytokine stimuli, as well as competition with pathogenic organisms for intraluminal nutrients (82, 83). Studies have also suggested that probiotics that include non-immune intestinal host defenses could strengthen tight junctions of the gut mucosa, increase mucous secretions, enhance motility, and produce amino acid by-products including arginine and glutamine, and SCFAs, that could secondarily function as protective foods for the gut (84–87).

A series of basic laboratory studies have highlighted the influence that probiotic bacteria and commensal bacteria may have on the maturation of intestinal macrophages/dendritic cells and the production of various cytokines (Table 2).

**Table 2:** Probiotic and prebiotic induction of regulatory T cells.

**Prebiotics**	**Probiotic|Commensal microbial effects**	**References**
FOS	↑*Bifidobacteria* genus ↑levels IL-10|macrophages|dendritic cells expressing TLR2 and TLR4	(88) (89)
GOS	↑*Bifidobacteria* genus ↑Tregs|↑levels IL-10|↓IL-6|↓IL-1β|↓TNF-α	(90) (91)
Inulin	↑*Bifidobacteria* genus|↑*Lactobacilli* genus|*Faecalibacterium prausnitzii*|*Eubacterium* spp. ↑levels IL-10|dendritic cells	(92) (93)
Resistant starch	↑Firmicutes phyum ↑Tregs|spleen|Peyer's Patches	(94) (95)

*In vitro* and *in vivo* studies have appraised the effects of probiotics on the prevention/development of large bowel cancer (i.e., specifically in the colon) (96). Epidemiological studies have associated large bowel cancers with genetic, environmental risk factors, including diet and the nature of the intestinal bacterial cohort (97). Probiotics used in animal models have been shown to reduce the occurrence of precancerous lesions observed in aberrant crypts (98, 99). *In vitro* experiments with probiotics have further suggested that the administration of these beneficial bacteria could reduce hypertension and lower serum cholesterol (100, 101). Furthermore, animal studies have also suggested that *Helicobactor pylori* infection in germ free murine models could be averted with the administration of *Lactobacilli* to dislodge *H. pylori* from the stomach and that cell attachment and invasion by enteropathogenic bacteria such as *Escherichia coli* and other gram-negative pathobionts can be inhibited with the use of *Lactobacillus acidophilus* containing probiotics (102). Interestingly, in a murine model of experimental uremia (103) intestinal macrophages were skewed toward a pro-inflammatory phenotype with reduced phagocytic activity with resultant bacterial translocation that triggered a local inflammatory response. The administration of a *Lactobacilli* LB probiotic reduced bacterial translocation by improving macrophage phagocytic activity.

### Probiotics human studies

The extensive body of clinical evidence that supports the use of probiotics in the prevention or treatment of gastrointestinal diseases have been administered in clinical trials with pediatric and adult patients (104–107). Formulations with probiotic bacteria include members from the bacterial genera, *Lactobacilli, Bifidobacteria*, and *Streptoccocus* (i.e., *Streptococcus thermophiles*) or the yeast, *S. boulardii* (108). Numerous studies report that probiotics provide efficacy for reducing the risk of developing Clostridium-Difficile-Associated-Diarrhea (109); preventing antibiotic associated diarrhea (110, 111); and Traveler's diarrhea (112, 113). Moreover in a systematic review study of outpatients, *S. boulardii* was reported efficacious in preventing antibiotic associated diarrhea (110). Hence probiotics can have beneficial effects on diarrheal conditions and related gastrointestinal symptoms. Further, evidence-based probiotic formulations can be administered to either prevent or reduce the severity of pathogenic bacteria triggered intestinal inflammations. Consequently, inflammatory bowel diseases (IBDs) can present with major clinical inflammatory associated complications. Anti-inflammatory pharmaceutical agents have been used extensively to ameliorate the chronic inflammatory responses that occur associated with IBDs. *In vivo* laboratory studies have reported success with probiotics used to prevent or reduce the inflammatory response associated with colitis (114). Although encouraging, additional studies are needed and warrants further focused research to make conclusive inferences on the efficacy of probiotics for ulcerative colitis, Crohn's Disease, and liver diseases (e.g., NAFLD) (115).

Mechanistically, it has been posited that formulas supplemented with probiotic bacteria could induce changes in the stool pattern that is bifidogenic and that it could mimic that observed with breast-fed infants (116, 117). Alternatively, in an early study with undernourished Peruvian infants, especially among non-breast fed children with a high encumbrance of diarrheal disease, the administration of *Lactobacilli* GG was reported associated with significantly fewer episodes of diarrhea (118). Clinical efficacy with probiotic formulations have been reported to reduce the number of episodes of diarrhea and rotavirus shedding among chronically infected infants admitted to hospital, young children as well as adults (119). Studies specifically with *Lactobacillus* GG administered as a treatment modality, during acute rotavirus infections with diarrhea has been reported and associated with higher titres of polymeric immunoglobulin A to the infections with rotavirus (119, 120). Additionally, significant experimental and clinical studies have reported the efficacy for reducing the incidence of neonatal necrotizing enterocolitis with the administration of *Lactobalcilli* and *Bifidobacteria* (121, 122). Increased efficacy of probiotics have recently been reported to significantly decrease the risk of developing clinical complications related with necrotizing enterocolitis and sepsis; decrease mortality and length of hospital stay; as well as promote neonatal weight increases in very low birth weight infants (123). Further that probiotics were more efficatious when administered with breast milk and or an infant formula, and consumed for <6 weeks, provided in a dose of ~10^9^ CFU/day and the probiotic formulation included multiple strains (123).

## Prebiotics

Prebiotics, have been defined as non-digestible food components (i.e., non-digestible carbohydrates), are important functional foods that potentiate the action of commensal/beneficial bacteria in the intestines [Table 2; (124)]. Hence the effectiveness of prebiotics is largely dependent on these substances eluding hydrolysis and absorption in the proximal small intestines so as to reach the large bowel (125). Once in the large bowel to be utilized selectively by the commensal group of bacteria (124). These include, Fructooligosaccharide (FOS), Galactooligosaccharide (GOS), inulin, dietary carbohydrates, and Xylooligosaccharide (XOS) are among the most commonly studied prebiotics in clinical studies (126, 127). Interestingly, oligosaccharides in human breast milk have been reported to represent the quintessential prebiotic, as they can readily facilitate the favored growth of the *Bifidobacteria* and *Lactobacilli* genera in the large bowel of neonates that have been exclusively breast-fed (128).

The chemical structures of prebiotics prevents metabolism and absorption in the small bowel and leads to bacterial fermentation reactions in the large bowel (i.e., specifically the colon) to form combustible gases, lactate, and SCFAs (i.e., acetate, propionate, butyrate) (129) that have been associated with health benefits (130).

### Prebiotics: *in vitro* and animal experimental data

Strong clinical evidence pertaining to the potential health benefits of prebiotics, result from *in vitro* and *in vivo* study models (131–133) *in vitro* studies have shown that individual specific bacterial species from the *Bifidobacteria* and *Lactobacilli* genera will ferment selected prebiotics as defined by the production of SCFAs in an acid environment (134). The mechanism of this selective activity involves factors that include the lowering of colonic pH and the production of metabolites that can inhibit pathobiont growth while simultaneously promoting the growth of probiotic bacteria and the production of antimicrobial effects (135, 136). Studies have shown the preferential administration of prebiotics over probiotics for the selected growth of bacteria in the large bowel (137, 138). For example, the incorporation of oligosaccharides in doses of 5–7 g/day may lead to the proliferation of certain types of bacteria that are generally considered to be beneficial (i.e., *Bifidobacteria, Lactobacilli*, non-pathogenic *E. coli* while decreasing *Bacteroidaceae*) to the detriment of pathobionts; this re-equilibration or intestinal homeostasis of the colonic biotope has been designated as the *prebiotic effect* (139).

The effect of prebiotics on the proliferation of specific bacterial classes is complex. The interactions with the intestinal microbiome cannot be easily explained by prebiotic compounds acting as exclusive substrates (140). Exploiting intestinal bacterial communities through the introduction of prebiotics has the capacity to indirectly influence beneficial immune responses (141). The production of SCFAs the major products of bacterial fermentation of prebiotics in the absence of oxygen modulates the concentration of SCFAs by down regulating pro-inflammatory mediators by intestinal macrophages (142).

SCFAs such as propionic acid and butyric acid have been reported to inhibit molecular induced expression of adhesion molecules, chemokine formation with concomitant suppression of monocyte/macrophage immune activities and neutrophil recruitment that in combination suggest an anti-inflammatory effect (142). This effect is articulated by butyrate, which is reported to suppress lipopolysaccharide and cytokine-promoted production of pro-inflammatory mediators including TNF-α, IL-6 and nitric oxide while enhancing the release of IL-10 an anti-inflammatory cytokine (143–145). Interestingly SCFAs have been documented to decrease the *in vitro* adherence of monocytes and lymphocytes to human umbilical vein endothelial cells (146). Furthermore, butyric acid interactions with monocytes, has also been shown to reduce the constitutive and IFN-γ-induced expression of lymphocyte function-associated antigen 3 and intercellular adhesion molecule-1 (147). In addition SCFAs regulate several functions expressed by leukocytes and these include production of a number of cytokines (i.e., TNF-α, IL-2, IL-6, and IL-10), eicosanoids and chemokines (e.g., macrophage chemo-attractant protein-1 and cytokine induce neutrophil chemo-attractant-2) (148).

SCFAs have also been reported to modulate the production of prostaglandin E2 (PGE2) and that this activity stimulates the *in vitro* production of PGE2 by human monocytes (149). This eicosanoid has been shown to suppress T cell receptor signaling and may play an important role in the resolution of inflammatory responses toward equilibrium (150), redefining it as an anti-inflammatory prostanoid by attenuating the formation of IL-1β and TNF-α by macrophages and Th1 differentiation (151). SCFAs such as acetic acid and propionic acid have been reported to reduce TNF-α formation induced by LPS that have been stimulated by human neutrophils (152). Moreover, reports relative the effects of propionate and butyrate demonstrate an inhibitory expression profile of pro-inflammatory mediators (i.e., TNF-α, CINC-2αβ, NO) from rat neutrophils through the attenuation of NF-kB activation (153). Prebiotics such as FOS, GOS, inulin and resistant starch have been demonstrated to affect microbial genera (indigenous *Bifidobacteria, Lactobacilli, Faecalibacteria*) (154, 155) that progress the up-regulation of Tregs by different mechanistic pathways and in turn re-regulate pro-inflammatory activity (94).

Prebiotics influencing immune modulation by exploiting the metabolic activities of commensal intestinal bacteria where experimental murine models suggest that such activity can significantly reduce the precancerous colonic lesions present as aberrant crypt foci (156). Other studies have documented that prebiotics augment the bioavailability and absorption of minerals such as calcium and may affect the metabolism of other minerals namely, magnesium, iron, and zinc (157); stimulating the reduction of endogenous carcinogens such as sialomucin (158); and reducing the growth of tumors in murine models of carcinogenesis (159).

## Prebiotics and human studies

Clinical studies have confirmed that FOS has a bifidogenic effect on the human large bowel (i.e., colon) and the endogenous intestinal microbiome (160, 161). Moreover, a number of clinical studies have also demonstrated the bifidogenic effect for inulin-type fructans (162). These studies have effectively demonstrated the growth promoting activity of prebiotics and the targeting of the bacterial genera *Lactobacilli* and *Bifidobacteria* (163). The overall implications from the clinical data is that the proposed bifidogenic effect is not simply attributed to prebiotics as preferential substrates for the commensal bacterial cohort, rather that prebiotic substances can interact with other commensal bacteria and may be subject to associations with environmental fluctuations including variations in luminal pH, and other unknown factors in order to achieve a net bifidogenic effect in the large bowel (164). In a clinical study with children attending day care daily supplementation with oligofructose (dose: 2 g/day over 3 weeks) was associated with significantly fewer episodes of diarrhea, flatulence, vomiting and fever, and with reductions in the level of pathobionts from the *Clostridia* and *Staphylococci* genera and with increases in *Bifidobacteria* genus (165). Infants supplemented with formula that included a prebiotic mixture (dose: 8 g/L) achieved normal growth and stool features that were more similar to those of breast-fed infants and in comparison with infants fed an un-supplemented formula (166). Others have reported no effect for oligofructose-supplemented infant cereal (167). Studies on the effect prebiotics have on the intestinal microbiome have reported that the consumption, daily, of whole-grain-wheat was observed to provide a pronounced prebiotic effect on the composition of the intestinal microbiota, positing that this activity may contribute toward beneficial physiological effects (168). Other studies have reported that prebiotics in the form of a whole-grain-maize augmented cereal (dose: 48/day) induced a bifidogenic modulatory effect on the intestinal microbiota (169). No significant changes were observed in serum lipid profiles, blood glucose levels or fecal output measures (169). Celiac disease is an autoimmune inflammatory problem characterized by the interplay between the host's genetic factors and gluten as the environmental trigger (170).

Clinical studies have posited and reported that the concentration of SCFAs in blood and more importantly in the intestines may predispose to or prevent pathological conditions such as IBD (171), cancer and obesity (172), diabetes (173), and symptoms such as diarrhea (174). Interestingly, in a study with healthy physically active subjects a synbiotic supplement, increased fecal *Lactobacillus paracasei* with no appreciable effect on mucosal immunity (175). This latter outcome is not surprising given that in healthy individuals mucosal immunity should be in equilibrium.

Clinical studies administering prebiotics to encourage intestinal microbiome shifts toward the production of increased levels of SCFAs remains contentious though with clinical studies, showing a benefit with beneficial microbial shifts and immune function (94, 176, 177) and others not (178).

## Bacteriophages

Bacteriophages are ancient dependent infective agents that have existed on this planet for millennia, were discovered about a century ago (179), and have been posited to participate in immunological activities of the host (180). The predominant intestinal phage load harbored by adult healthy individuals has been reported to be a member of the order *Caudovirales*, a double stranded DNA bacteriophage (from the families *Podoviridae, Siphoviridae*, and *Myoviridae*), single stranded DNA bacteriophages (from the families *Microviridae* and *Inoviridae*) and RNA viruses (181–184). Viruses that reside in the intestines are comprised predominantly of bacteriophages (including prophages) and to a lesser degree eukaryotic viruses, reported to be stably integrated into bacterial genomes (parasitized phase) and lytic phages which can infect and lyse bacteria and hence release virus particles (185). Bacteriophages can hence influence the bacterial structure of the microbiome through a parasitic or lytic phase of bacterial cells and show the greatest abundance and diversity in the intestines of healthy adults (186).

Recent reports show that the neonate is exposed to a diverse range of bacteriophages at birth (187), an exposure that together with bacteria could herald a further consideration of how immunological and metabolic tolerance is achieved in early life. Bacteriophage directed control of behaviors of how bacteria colonize and survive in different anatomical sites encourage developmental properties that helps establish commensal populations that reduces the risk of disease (182). Studies report that bacteriophages may be involved in important functions in human immunity by defending the intestinal epithelial barrier and mucosal tissue from infections by pathobionts (188).

Bacteriophages influencing the stability of the intestinal microbiome cohort indirectly shape the immunological and metabolic functions of intestinal immunity, especially as reported for the direct action on T and B cells (189). Bacteriophages have been reported to adhere to the mucus layer of the intestines, and that the interaction with glycan residues from mucin glycoproteins affords the viruses a niche that is in close proximity to the intestinal epithelial layer and mucosal surfaces (190). Furthermore, it is reported that when phages exhibit muco-adherent properties, such actions can influence the activity of the innate and cell mediated immune systems (191). Moreover, temperate phages that positively influence host immunity could do so by selective screening of the commensal bacterial cohort (191).

In concert with peptides that exhibit antimicrobial properties in the mucus layer there is demonstrated a control over the density of the commensal bacteria that can occupy the mucus layer. This effective local control provides a protective barrier against commensal pathobionts while establishing a symbiotic relationship with the host; an immunological defense mechanism not derived from the host.

In the pre-antibiotic era, the use of bacteriophages to treat infections was an early example of the immunogenicity effect that was attributed to bacteriophages (192). A number of studies have documented the immune-modulatory effects of bacteriophages. These have included, (i) an anti-bacteriophage immune response, whereby exposure to bacteriophages in the circulation have induced strong anti-bacteriophage humoral responses resulting in swift and proficient neutralization and clearance of the phage on subsequent exposures to the virus (193, 194); (ii) bacteriophage-mediated re-regulation of the over-production of reactive oxygen species by phagocytes is a critical immunological effect that significantly contributed to the favorable effects of bacteriophage therapy in patients diagnosed with a life-threatening condition such as sepsis (193); and iii) a chemically modified phage with high affinity ligands for cell specific receptors has been reported to induce humoral and cellular immune responses regressing solid tumors in murine models (193, 194).

Certain Eastern European countries with bacteriophage treatment centers, routinely administer phage therapies for the prophylactic and therapeutic treatment of bacterial infections for ulcers/wounds, septicemia, UTIs, MRSA and others (195). Reports note though that the state of the immune system can determine what type of an effect the virome may have that then determines the interactions that ensue with host immunity. This is particularly relevant with inflammatory diseases such Crohn's Disease and Ulcerative colitis where the intestinal virome has been deemed to be abnormally altered in terms of increased bacteriophage richness and then correlated to decreased bacterial diversity (196). Moreover the bacteriophage changes were Inflammatory Bowel Disease (IBD) specific. Such specific effects have been consistent with studies that have reported decreases in diversity and abundance of the bacterial phyla (197). Specifically decreased *Bacteroidetes* and *Firmicutes* phyla from the fecal samples, were reported associated with IBD (197).

It is hence very probable that bacteriophages may control and cull the bacterial population in the intestines with substantial turnover that as such significantly influences bacterial diversity/abundance and metabolism in the intestines. Furthermore, increases in the intestinal virome due to decreases in commensal bacterial diversity and abundance could also explain the inefficacy that has been reported with certain probiotic treatments for IBD, especially in patients diagnosed with Crohn's Disease.

## Summary

The commensal microbial cohort functions to develop and establish the host's immune system (i.e., mucosal and cell mediated) in order to promote immunological and metabolic tolerance. The microbiome complement effect is mediated by bacteriological factors that stimulate cells of the host; and these factors operate across a diverse set of host receptors and cellular molecular targets that are expressed on the surface or within the cells of the host. The host receptors that interact with microbial factors include numerous operators such as pattern recognition receptors (i.e., TLRs), receptors such as C-type lectin and nucleotide oligomerization and RIG-1-like receptors, which can sense important microbial macromolecular constituents such as nucleic acids (i.e., DNA, RNA), proteins and cell wall components (198).

Specifically, probiotics and prebiotics are reported to have positive immuno-equilibrium restorative effects. An increasingly supported posit is that bacteria such as those from the probiotic genera of *Bifidobacteria* and *Lactobacilli* can participate in immune-regulation and do so by inducing regulatory T cells (199, 200). The beneficial immune-modulatory effects are elicited across several molecules, that include microbial cell walls, peptidoglycan, and exopolysaccharides, through interactions with specific host cell receptors (i.e., Toll-Like Receptor (TLR)-2 and TLR-4) (200). Relative to prebiotics, these compounds encourage the intestinal microbiome production of SCFAs, which have a central role in intestinal immunogenicity (201). It is generally accepted that SCFAs such as acetate propionate and butyrate can interact with local intestinal epithelial and mucosal immune tissues as well as having significant epigenetic effects (202), and are essential participants in health and disease. Bacteriophages seem to have a two-way role in the intestines that balances health and disease; from determinants of decreased abundance and diversity of the commensal microbiome to the control of bacterial diversity and abundance as a necessary factor to control pathobiont insults that balances the risk of disease and health. Bacteriophages, it would seem display immune-suppressive characteristics in the intestines across involvement in a number of immune related areas such as the control of inflammation and autoimmune reactions (189).

## Author contributions

LV conception and design of the manuscript. LV, GV, and SH read, amended, and approved the final version of the manuscript.

### Conflict of interest statement

LV has received National Institute of Complementary Medicine and National Health and Medical Research Council of Australia competitive funding and Industry support for research into probiotics. LV and SH participate in research on probiotics in Medlab Clinical's research laboratory facility in Sydney, Australia. The remaining author declares that the research was conducted in the absence of any commercial or financial relationships that could be construed as a potential conflict of interest.
